# Genetics of the thrombomodulin-endothelial cell protein C receptor system and the risk of early-onset ischemic stroke

**DOI:** 10.1371/journal.pone.0206554

**Published:** 2018-11-01

**Authors:** John W. Cole, Huichun Xu, Kathleen Ryan, Thomas Jaworek, Nicole Dueker, Patrick McArdle, Brady Gaynor, Yu-Ching Cheng, Jeffrey O'Connell, Steve Bevan, Rainer Malik, Naveed Uddin Ahmed, Philippe Amouyel, Sheraz Anjum, Joshua C. Bis, David Crosslin, John Danesh, Stefan T. Engelter, Myriam Fornage, Philippe Frossard, Christian Gieger, Anne-Katrin Giese, Caspar Grond-Ginsbach, Weang Kee Ho, Elizabeth Holliday, Jemma Hopewell, M. Hussain, W. Iqbal, S. Jabeen, Jim Jannes, Ayeesha Kamal, Yoichiro Kamatani, Sandip Kanse, Manja Kloss, Mark Lathrop, Didier Leys, Arne Lindgren, W. T. Longstreth, Khalid Mahmood, Christa Meisinger, Tiina M. Metso, Thomas Mosley, Martina Müller-Nurasyid, Bo Norrving, Eugenio Parati, Annette Peters, Alessandro Pezzini, I. Quereshi, Asif Rasheed, A. Rauf, T. Salam, Jess Shen, Agnieszka Słowik, Tara Stanne, Konstantin Strauch, Turgut Tatlisumak, Vincent N. Thijs, Steffen Tiedt, Matthew Traylor, Melanie Waldenberger, Matthew Walters, Wei Zhao, Giorgio Boncoraglio, Stéphanie Debette, Christina Jern, Christopher Levi, Hugh Markus, James Meschia, Arndt Rolfs, Peter Rothwell, Danish Saleheen, Sudha Seshadri, Pankaj Sharma, Cathie Sudlow, Bradford Worrall, METASTROKE Consortium of the ISGC, WTCCC-2 Consortium, O. Colin Stine, Steven J. Kittner, Braxton D. Mitchell

**Affiliations:** 1 Veterans Affairs Maryland Health Care System; University of Maryland School of Medicine, Baltimore, MD, United States of America; 2 University of Maryland School of Medicine, Baltimore, MD, United States of America; 3 University of Miami, Miami, Florida, United States of America; 4 Food and Drug Administration, White Oak, MD, United States of America; 5 University of Lincoln, Lincoln, United Kingdom; 6 Klinikum der Universität München, Munich, Germany; 7 Liaquat National Hospital, Karachi, Pakistan; 8 Inserm, Lille, France; 9 Center for Non-Communicable Diseases, Karachi, Pakistan; 10 University of Washington, Seattle, WA, United States of America; 11 University of Cambridge, Cambridge, United Kingdom; 12 University Hospital Basel, Basel, Switzerland; 13 University of Texas Health Science Center at Houston, Houston, TX, United States of America; 14 Helmholtz Zentrum München, München, Germany; 15 Massachusetts General Hospital, Boston, MA, United States of America; 16 Heidelberg University, Heidelberg, Germany; 17 University of Newcastle, Newcastle, Australia; 18 University of Oxford, Oxford, United Kingdom; 19 Lahore General Hospital, Lahore, Pakistan; 20 University of Adelaide, Adelaide, Australia; 21 Aga Khan University Hospital, Karachi, Pakistan; 22 RIKEN Center for Integrative Medical Sciences, Yokohama City, Kanagawa, Japan; 23 Institute of Basic Medical Sciences, Oslo, Norway; 24 McGill University and Québec Innovation Centre, Montreal, Canada; 25 University of Lille; INSERM, Lille, France; 26 Lund University, Lund, Sweden; 27 Harborview Medical Center, Seattle, WA, United States of America; 28 Dow University of Health Sciences, Civil Hospital, Karachi, Pakistan; 29 Central Hospital of Augsburg, Augsburg, Germany; 30 Helsinki University Central Hospital, Helsinki, Finland; 31 University of Mississippi Medical Center, Jackson, MS, United States of America; 32 Institute of Medical Informatics, Ludwig-Maximilians-University, Munich, Germany; 33 Fondazione IRCCS Istituto Neurologico, Milan, Italy; 34 GSF-National Research Center for Environment and Health, Munich, Germany; 35 Universita Degli Studi di Brescia, Brescia, Italy; 36 King Edward Medical University and Mayo Hospital, Lahore, Pakistan; 37 Lunenfeld Tenubaum Research Institute, Toronto, Ontario, Canada; 38 Jagiellonian University Medical College, Krakow, Poland; 39 Institute of Biomedicine, Gothenburg, Sweden; 40 Ludwig-Maximilians University Munich, Munich, Germany; 41 Helsinki University Central Hospital, Helsinki, Finland; 42 Florey Institute of Neuroscience and Mental Health, University of Melbourne, Melbourne, Australia; 43 Institute for Stroke and Dementia Research, Ludwig-Maximilians Universität München, Munich, Germany; 44 University of Glasgow, Glasgow, Scotland; 45 Translational Medicine and Human Genetics, Philadelphia, PA, United States of America; 46 Bordeaux University, Bordeaux, France; 47 Institute of Biomedicine, Gothenburg, Sweden; 48 John Hunter Hospital, New Lambton Heights, NSW, Australia; 49 Mayo Clinic, Jacksonville, FL, United States of America; 50 University of Rostock, Rostock, Mecklenburg-Vorpommern, Germany; 51 John Radcliffe Hospital, Oxford, United Kingdom; 52 University of Pennsylvania, Philadelphia, PA, United States of America; 53 Boston University School of Medicine, Boston, MA, United States of America; 54 Royal Holloway, University of London, London, United Kingdom; 55 University of Edinburgh, Edinburgh, Scotland; 56 University of Virginia, Charlottesville, VA, United States of America; Seoul National University Hospital, REPUBLIC OF KOREA

## Abstract

**Background and purpose:**

Polymorphisms in coagulation genes have been associated with early-onset ischemic stroke. Here we pursue an *a priori* hypothesis that genetic variation in the endothelial-based receptors of the thrombomodulin−protein C system (*THBD* and *PROCR*) may similarly be associated with early-onset ischemic stroke. We explored this hypothesis utilizing a multi-stage design of discovery and replication.

**Methods:**

Discovery was performed in the Genetics-of-Early-Onset Stroke (GEOS) Study, a biracial population-based case-control study of ischemic stroke among men and women aged 15–49 including 829 cases of first ischemic stroke (42.2% African-American) and 850 age-comparable stroke-free controls (38.1% African-American). Twenty-four single-nucleotide-polymorphisms (SNPs) in *THBD* and 22 SNPs in *PROCR* were evaluated. Following LD pruning (r^2^≥0.8), we advanced uncorrelated SNPs forward for association analyses. Associated SNPs were evaluated for replication in an early-onset ischemic stroke population (onset-age<60 years) consisting of 3676 cases and 21118 non-stroke controls from 6 case–control studies. Lastly, we determined if the replicated SNPs also associated with older-onset ischemic stroke in the METASTROKE data-base.

**Results:**

Among GEOS Caucasians, *PROCR* rs9574, which was in strong LD with 8 other SNPs, and one additional independent SNP rs2069951, were significantly associated with ischemic stroke (rs9574, OR = 1.33, p = 0.003; rs2069951, OR = 1.80, p = 0.006) using an additive-model adjusting for age, gender and population-structure. Adjusting for risk factors did not change the associations; however, associations were strengthened among those without risk factors. *PROCR* rs9574 also associated with early-onset ischemic stroke in the replication sample (OR = 1.08, p = 0.015), but not older-onset stroke. There were no *PROCR* associations in African-Americans, nor were there any *THBD* associations in either ethnicity.

**Conclusion:**

*PROCR* polymorphisms are associated with early-onset ischemic stroke in Caucasians.

## Introduction

**Fig 1:**
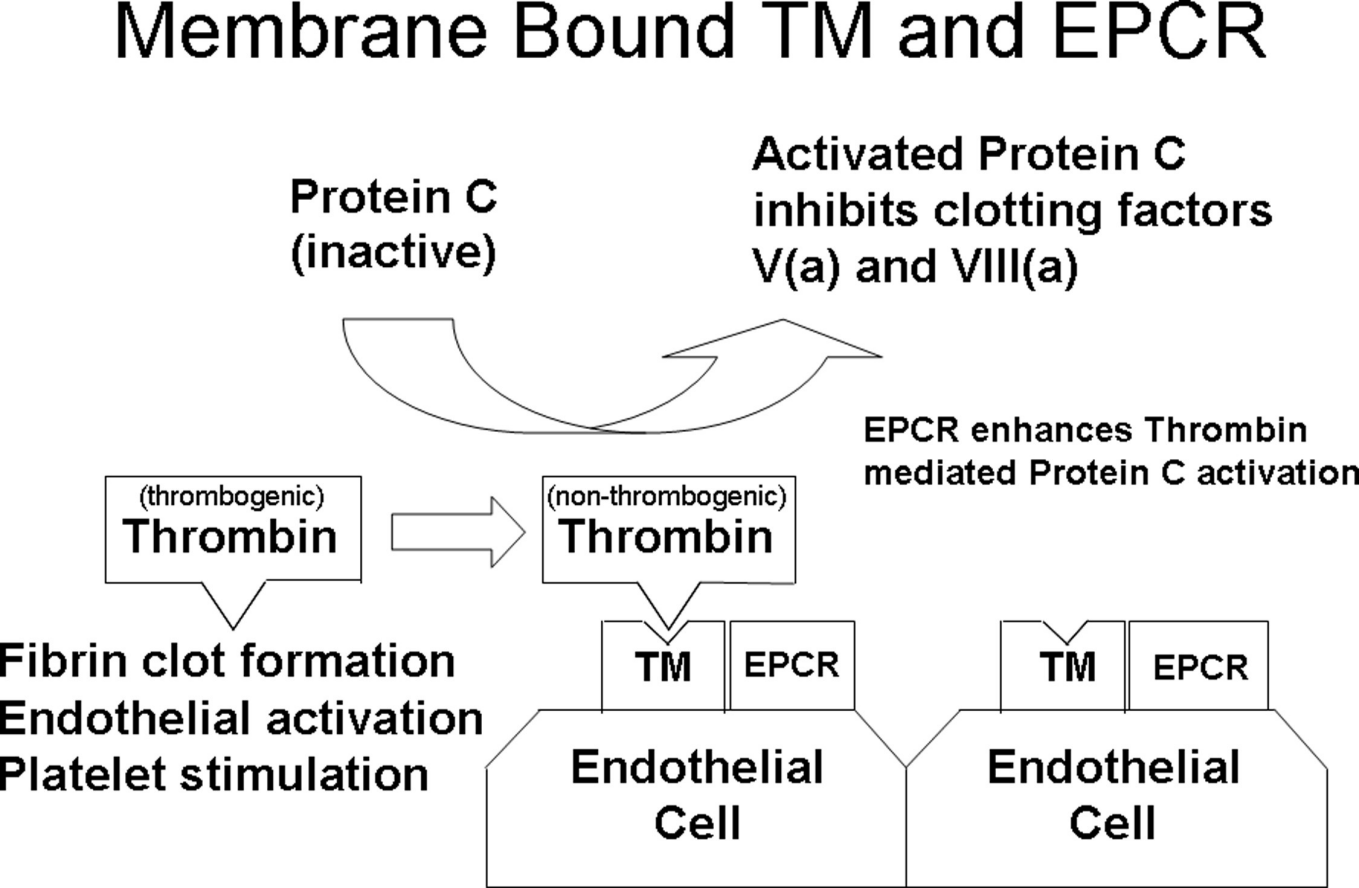
The thrombomodulin−protein C receptor (TM-EPCR) system located on the endothelial surface.

Hemostasis is a dynamic balance between factors that promote clot formation and factors that promote antithrombotic activity and/or fibrinolysis. Central to this balance is the thrombomodulin-protein C antithrombotic system that is located on the endothelial surface, which plays a key role in regulating both coagulation and inflammation. Thrombomodulin forms a 1:1 complex with thrombin on the vascular endothelium, thereby inhibiting the procoagulant actions of thrombin and converting protein C to activated protein C [1]. Activated protein C promotes fibrinolysis, inhibits thrombosis by inactivating coagulation factors Va and VIIIa, and reduces inflammation by decreasing white blood cell and nuclear factor kappa-B activation [2–5]. The activation of protein C by the thrombin-thrombomodulin complex is enhanced when the substrate protein C is presented by the endothelial cell protein C receptor. These relationships are demonstrated in **Fig 1**. Given the central role that the thrombomodulin-protein C pathway plays in thrombosis and inflammation, the genes encoding these receptor proteins are promising stroke susceptibility candidate genes. Prior genetic studies across the cardiovascular disease (CVD) spectrum have demonstrated increased risk in younger (vs. older) patients [6], including thrombosis [7]. Variants in other prothrombotic genes have also previously been associated with ischemic stroke, again, more consistently with early-onset versus later-onset disease [8,9,10]. As such, an *a priori* hypothesis to evaluate these 2 genes in the setting of ischemic stroke was developed and successfully funded. To this end we tested the hypothesis that *THBD* (OMIM 188040) and *PROCR* (OMIM 600646) variants are associated with early-onset ischemic stroke using a 2-stage discovery and replication design, and then addressed whether the identified variants also associated with older-onset disease.

## Methods

### Discovery population

The Genetics of Early Onset Stroke (GEOS) Study is a population-based case-control study designed to identify genes associated with early-onset ischemic stroke and to characterize interactions of identified stroke genes and/or SNPs with environmental risk factors. Participants (921 stroke cases and 941 controls) were recruited from the greater Baltimore-Washington area over 4-time periods between 1992–2008 [11]. The population is primarily composed of two self-reported ethnic groups, European-Americans (Caucasians) (EA; 54.5%) and African-Americans (AA; 40.4%), with the remaining 5.1% of individuals comprising other ethnicities including Chinese, Japanese, other Asians, and other unspecified. Stroke cases were hospitalized with a first cerebral infarction identified by discharge surveillance from one of the 59 hospitals in the greater Baltimore-Washington area and direct referral from regional neurologists. Cases were enrolled in either the sub-acute or chronic post-stroke phases as based on previously described case identification and enrollment procedures [8,11]. Ischemic strokes with the following characteristics were excluded from participation: stroke occurring as an immediate consequence of trauma; stroke within 48 hours after a hospital procedure, stroke within 60 days after the onset of a non-traumatic subarachnoid hemorrhage, and cerebral venous thrombosis. The abstracted hospital records of cases were reviewed and adjudicated for ischemic stroke subtype by a pair of neurologists per previously published procedures [12,13], with disagreements resolved by a third neurologist. The ischemic stroke subtype classification system retains information on all probable and possible causes, and is reducible to the more widely used TOAST system [14] that assigns each case to a single category. All cases had age of first stroke between 15–49 years and were recruited within three years of stroke.

For these genetic analyses, we included only Caucasians and African-Americans, and excluded cases with known single-gene or mitochondrial disorders recognized by a distinctive phenotype (e.g. cerebral autosomal dominant arteriopathy with subcortical infarcts and leukoencephalopathy (CADASIL), mitochondrial encephalopathy with lactic acidosis and stroke-like episodes (MELAS), homocystinuria, Fabry disease, or sickle cell anemia). Additional exclusions included: mechanical aortic or mitral valve at the time of index stroke; untreated or actively treated bacterial endocarditis at the time of the index stroke; neurosyphilis or other CNS infections; neurosarcoidosis; severe sepsis with hypotension at the time of the index stroke; cerebral vasculitis by angiogram and clinical criteria; post-radiation arteriopathy; left atrial myxoma; major congenital heart disease; and cocaine use in the 48 hours prior to their stroke.

Control participants without a history of stroke were identified by random-digit dialing. Controls were balanced to cases by age and region of residence in each study period and were additionally balanced for race/ethnicity in the latter two participant collection periods.

Traditional stroke risk factors and other study variables, including age, race/ethnicity, history of hypertension, diabetes, myocardial infarction (MI) and current smoking status (defined as use within one month prior to event for cases and at a comparable reference time for controls), were also collected during a standardized interview. Age, race/ethnicity, and cigarette smoking status were determined by subject reports (or proxy report, if a participant was unable to answer). Hypertension, diabetes mellitus, and MI were determined by asking study participants (or a proxy) whether a physician had ever told them that they had the condition. This study was conducted with the consent of all study subjects and was approved by the University of Maryland at Baltimore Institutional Review Board.

### Genotyping

Genomic DNA was isolated from a variety of sample types, including cell line (55.2%), whole blood (43.1%), mouthwash (0.4%) and buccal swab (0.05%). Whole genome amplification (Qiagen REPLI-g kit, Valencia, CA, USA) was used to obtain sufficient DNA for genotyping in 1.3% of samples. The genotype data implemented in this study was obtained from two fixed-content SNP panels developed by Illumina (Illumina, San Diego, CA, USA), a genome-wide association (GWA) genotyping array, the HumanOmni1-Quad_v1-0_B BeadChip, and a cardiovascular disease (CVD) SNP panel, the ITMAT-Broad-CARe array, that included *THBD* and *PROCR*. Genotyping quality from both arrays was excellent with individual SNP call rates > 98% and a between-panel concordance rate of 99.996% based on study duplicates (for further details please see **S1 File**) [11].

### SNP Selection and inclusion criteria

From each array, we extracted all SNPs in the *THBD* (chr 20: 22,974,270–22,978,301 bp) and *PROCR* (chr 20: 33,223,435–33,228,826 bp) genes (NCBI Build 37) and then added all additional SNPs within 10kb upstream and downstream to capture regulatory regions. In total, we identified 24 SNPs in *THBD* (17 GWA; 18 CVD; 11 overlap) and 22 SNPs in *PROCR* (22 GWA; 4 CVD; 4 overlap). Across both genes there were nine SNPs unique to European-Americans (EA; Caucasians) and six SNPs unique to African-Americans. There was an overlap of 15 SNPs between the two genotype sources with an average SNP call concordance rate between platforms of >99%. Other predefined selection and quality control criteria included required; 1) HWE p-values >0.01, 2) Call rate > 98%, and MAF >0.01 in race-stratified samples.

### Analyses

Genetic association analyses were performed using the PLINK statistical software program [15]. Prior to the association analysis we pruned the genotyped SNPs on the basis of linkage-disequilibrium (LD), such that for any SNPs in high LD (r^2^≥0.8) we retained only a single representative SNP. This LD pruning was performed within each ethnic group separately using PLINK. Within each ethnic group separately, we then used an additive logistic regression model to test for association of genotype with stroke, adjusting for age and gender, and population structure (principal components from GWAS array or CVD panel). Secondary analyses were performed to determine if any observed associations were more prominent in those with cardiovascular risk based on the presence of the traditional risk factors as described above and previously [11]. For all association analyses, we defined a significant Boferroni-corrected p-value as p<0.05 divided by the number of gene- and ethnicity-specific independent (LD-pruned) SNPs (i.e., p = 0.05 / # independent LD-Pruned SNPs).

### Replication and extension to older onset stroke

We sought to replicate any associated SNPs identified in the GEOS Study in an independent set of early-onset stroke studies (the Genetics of Early Onset Stroke Consortium) previously reported by Cheng et al. [16] after excluding the GEOS samples from the replication set, as meta-analyzed implementing the GWAMA program. The studies included in the replication were: CADISP, Cervical Artery Dissection and Ischemic Stroke Patients [17]; MILANO, Besta Stroke Study; RACE, Risk Assessment of Cerebrovascular Events Study; SIFAP, Stroke in Young Fabry Patients; and WTCCC2, Wellcome Trust Case–Control Consortium 2 [16]. The details of each of replication cohort are available in the supplementary data of Cheng et al. [16]. In short, only confirmed ischemic strokes, first ever or recurrent, were included in these studies, TIAs and hemorrhagic strokes were excluded. SNPs whose associations replicated in the Genetics of Early Onset Stroke Consortium were then tested for association with later- or older-onset stroke via *in silico* lookup in the METASTROKE Consortium [17]; the mean age of stroke onset ranged from 57.3–81.6 years among the 14 contributing cohorts of METASTROKE (not including GEOS). Further details regarding the data collection, organization, and relationships between METASTROKE and the other studies involved can be found in the **S1 File** and **S1 Dataset**.

The aggregated data that support the findings of this study are available from the corresponding author and participating studies upon reasonable request as listed in the **S1 Dataset**. Further, each study can be contacted to attain their data individually, and for the NIH funded studies, study data is available via request from the database of Genotypes and Phenotypes (dbGaP) @ https://www.ncbi.nlm.nih.gov/gap/.

## Results

Characteristics of the young-onset stroke discovery and replication studies are provided in **Table 1**. After exclusions, the GEOS Discovery Stage included 448 ischemic stroke cases (mean age stoke-onset = 41.0 yrs) and 498 controls of EA ancestry, and 381 ischemic stroke cases (mean age stroke-onset = 41.9 yrs) and 352 controls of AA ancestry. Further demographic and risk factor characteristics by case–control status for the GEOS Discovery Stage are described in **Table A in S1 File**.

**Table 1:** Characteristics of participating studies.

Study	Cases			Controls				Ancestry	Country
	Subjects, n	Age, mean (SD)	Male, n (%)	Subjects, n	Age, mean (SD)	Male, n (%)	External Control		
**Stage 1: Discovery Stage**									
GEOS EA	448	41.0 (7.0)	275 (61.4)	498	39.5 (6.7)	282 (56.6)	No	EA	USA
GEOS AA	381	41.9 (6.8)	207 (54.3)	352	40.0 (6.8)	196 (55.7)	No	AA	USA
Total	829			850					
**Stage 2: Replication Stage**									
CADISP	555	43.7 (9.9)	339 (61.1)	9259	N/A	N/A	No	EA	Belgium, France, Germany, Italy, Switzerland, and Finland
MILANO	201	45.0 (10.4)	120 (60.9)	407	50.8 (8.1)	357 (87.8)	No	EA	Italy
RACE 1	1218	50.1 (9.9)	638 (52.4)	1158	51.9 (7.9)	613 (53)	PROMIS	South Asian	Pakistan
RACE 2	339	50.2 (9.2)	272 (80.4)	3295	60.9 (13.2)	1838 (55.8)	PROMIS	South Asian	Pakistan
SIFAP	981	41.7 (7.4)	599 (61.1)	1824	55.2 (11.6)	899 (49.3)	KORA	EA	Germany
WTCCC2-UK	382	51.9 (7.3)	228 (59.7)	5175	52	2611 (50.5)	British Birth Cohort and UK Blood Service Control	EA	UK
Total	3,676			21,118					

AA indicates African ancestry; CADISP, Cervical Artery Dissection and Ischemic Stroke Patients; EA, European ancestry; GEOS, Genetics of Early-Onset Stroke; MILANO, Besta Stroke Study; RACE, Risk Assessment of Cerebrovascular Events Study; SIFAP, Stroke in Young Fabry Patients; and WTCCC2, Wellcome Trust Case-Control Consortium 2.

LD pruning resulted in 13 *THBD* SNPs in EAs and 13 *THBD* SNPs in AAs, and an additional 4 *THBD* SNPs in EAs on the CVD chip; and 5 *PROCR* SNPs in EAs and 11 *PROCR* SNPs in AAs (see **Table 2**and **Table B in S1 File**).

**Table 2:** PROCR association results of lead LD SNPs in Discovery GEOS European-Americans for all-stroke detailing allelic variants, effect allele frequencies (EAF) and as stratified by the absence or presence of vascular risk factors, and lead LD SNP young-onset replication results.

		Discovery: GEOS												Replication: Young-Onset Stroke Cohort without GEOS (Meta-analysis of 6 studies)			
			448 cases	498 controls	Primary Model (adjusted for age and gender)			0 Risk Factors (167 cases / 315 controls)			≥ 1 Risk Factors (273 cases / 183 controls)			3671 Cases / 21119 Controls			
rsID*	LD_SNPs	BP_ Build37	EAF	EAF	OR	95%CI	P	OR	95%CI	P	OR	95%CI	P	OR	95%CI	P	EAF
rs9574 (**C**/G) (intronic)	rs945960 (intronic) rs1415774 (intronic) rs2069952 (intronic) rs6088753 (intronic) rs2378337 (intronic) rs6087683 (intronic) rs2065979 (intronic) rs6088747 (intronic)	33764632	0.49	0.41	1.33	1.11–1.61	**0.003**	1.50	1.14–1.99	**0.0046**	1.23	0.94–1.61	0.1367	1.08	1.02–1.16	**0.015**	0.397
rs2069951 (**G**/A) (intronic)	NA	33763764	0.96	0.93	1.80	1.18–2.75	**0.006**	4.82	2.14–10.89	**0.0002**	0.80	0.43–1.52	0.4870	1.08	0.93–1.27	0.331	0.956
rs6087682 (**A**/T) (intronic)	rs6060278 (intronic)	33752897	0.80	0.75	1.26	1.01–1.58	0.050	1.33	0.96–1.87	0.0910	1.14	0.82–1.6	0.4510	1.03	0.97–1.12	0.368	0.766
rs867186 (**A**/G) (missense)	rs7265317 (intronic) rs11907011 (intronic) rs8119351 (intronic)	33764554	0.91	0.90	1.18	0.86–1.64	0.311	1.63	1–2.69	0.0533	0.80	0.5–1.32	0.3789	1.10	0.98–1.26	0.133	0.865
rs1415775 (**C**/T) (intronic)	NA	33765771	0.76	0.74	1.09	0.89–1.35	0.428	1.01	0.74–1.38	0.9694	1.23	0.92–1.67	0.1773	0.99	0.92–1.07	0.745	0.759

*EAF = Effect allele bolded

Association analyses of the EA revealed a significant association of *PROCR* rs9574 with ischemic stroke, with the rs9574C allele (MAF case/control = 0.49/0.41) associated with a 1.33-fold increased odds of stroke compared to the G allele (p = 0.003; **Table 2**). Another independent *PROCR* SNP rs2069951 was also associated with ischemic stroke significantly in EA (OR = 1.80, P = 0.006). None of the *PROCR* SNPs were associated with stroke in AA, nor were any associations observed between *THBD* SNPs and stroke in either ethnic group. An exploratory analyses of stroke subtypes (e.g., TOAST-defined large artery (LA), small artery, cardioembolic, and cryptogenic) did not reveal significant associations with any variants, although the sample sizes were small (range: 33 LA to 230 cryptogenic) in EAs.

To further characterize the EA *PROCR* associations, we performed a secondary analysis to evaluate the impact of concomitant vascular risk factors. We repeated the association analysis with additional adjustment for vascular risk factors (i.e., hypertension, diabetes mellitus, angina/MI, and current cigarette smoking) and found the association results to be essentially unchanged (data not shown). However, when stratifying for the presence or absence of each vascular risk factor, we observed a stronger, but non-statistically significant, association of rs9574 and rs2069951 with stroke in the absence of each risk factor when considered separately, with the direction of association similar across each risk factor (data not shown). To obtain a more comprehensive picture, we therefore compared subjects with zero vascular risk factors to those with at least one vascular risk factor. In the subset of EA participants without vascular risk factors (n = 167 cases and 315 controls), both SNPs were more strongly associated with stroke (rs9574, OR = 1.50, p = 0.0046; rs2069951, OR = 4.82, p = 0.0002; see **Table 2**).

### Replication of the PROCR rs9574 association with early onset stroke

We sought to replicate the *PROCR* rs9574 and rs2069951 association in the Early-Onset Stroke Consortium [16], with exclusion of the GEOS study. This replication sample included 3,676 cases and 21,118 controls. Only rs9574 replicated; the effect allele frequency of rs9574C was 0.39, with association analyses demonstrating an OR of 1.08 (p = 0.015) (**Table 2**). The ischemic stroke replication results of the LD-pruned *PROCR* SNPs among Caucasians in the young-onset stroke consortium, inclusive and exclusive of GEOS, are shown in **Table C in S1 File**. There was no significant correlation between stroke subtypes and *PROCR* rs9574 in the replication samples.

### Older-onset stroke

To determine if *PROCR* rs9574 and/or the other previously identified LD-pruned SNPs were associated with older-onset stroke, these SNPs were evaluated in the METASTROKE cohort [18]. Lookups found no replication of these SNPs with ischemic stroke or in any subtype (data not shown).

## Discussion

We observed a significant association between *PROCR* rs9574 and early-onset ischemic stroke that replicated in a large independent sample of early-onset ischemic stroke. Prior studies have demonstrated that mutations in *PROCR* have been associated with venous thromboembolism (VTE) [19] and myocardial infarction [20,21], as well as with late fetal loss during pregnancy [22]. Specific to ischemic stroke, *PROCR* associations have been inconsistently reported [23, 24], which may in part be related to variations in the age and ethnicity of the populations evaluated. Our study is the first to specifically identify and replicate *PROCR* associations in a young-onset ischemic stroke population of European descent. Our failure to detect an association in GEOS AA may reflect low power and/or that a true causal variant is not well tagged in African-Americans. Our findings add to the growing evidence that prothrombotic mechanisms may be more important for younger compared to older onset stroke as demonstrated with other established prothrombotic variants including Prothrombin G20210A [8], Factor XI [10] and Factor V Leiden [25]. This is also in line with the lack of association we observed between *PROCR* rs9574 and older onset stroke in the METASTROKE lookup. Our findings are also consistent with the hypothesis that *PROCR* (and perhaps by analogy other thrombosis-related genes) may be more relevant, or easier to detect, in the setting of a paucity of standard vascular risk factors, as these factors may induce risk via non-thrombotic mechanisms. This may again also partially explain why we did not see replication in the older-onset METASTROKE population, which also has a greater vascular risk factor burden. Differing genetic mechanisms may also partially explain why African-Americans did not demonstrate the associations seen in their Caucasian counterparts.

Strengths of our study include the well-phenotyped and relatively large young-onset discovery sample size, as well as the large replication sample. Notably, GEOS cases are part of the METASROKE as were other young-onset strokes, yet despite the inclusion of the GEOS samples, there was no association seen in this primarily older-onset cohort. A potential study limitation relates to the replication sample, which is predominantly of European rather than North-American origin, although the MAFs were roughly similar on both sides of the Atlantic. Another limitation is that our discovery population-based design, with recruitment at over 50 regional hospitals, precluded consistent assessment of the presence of patent foramen ovale (PFO) and potential paradoxical embolism among cases, given *PROCR genetics* are known to increase the risk of venous thromboembolism. This is important because an established mechanism by which *PROCR* variation could cause ischemic stroke is via venous thrombosis and paradoxical embolization. Our study was also limited to non-fatal ischemic strokes, so the possibility that our findings are due to a survival bias cannot be ruled out; though this is unlikely given the low case-fatality rate in this population [26]. Another limitation is that our study provides no information about the role of *PROCR* in ischemic stroke among young adults with a personal or family history of prior early-onset thrombotic events. Lastly, while some intronic mutations can affect gene expression levels by introducing novel splice sites, activating novel promoters (which may direct sense or antisense transcription causing alterations in mRNA, miRNA or lncRNA expression), or by introducing/eliminating enhancer activity, our study does not provide any such detailed mechanistic analyses. Despite these shortcomings, we have identified several *PROCR* variants in strong LD that associate and replicate with ischemic stroke among young Caucasians. While these findings are interesting, it is too early to assess their clinical implications regarding anticoagulation and/or genetic testing, as examples. Further replication and research are required to better understand these findings.

## Conclusion

*PROCR*, *but not THBD*, polymorphisms are associated with early-onset ischemic stroke in young Caucasians.

## Supporting information

S1 File Supplementary information. (Table A) GEOS Characteristics by case–control status. (Table B) Results of linkage-disequilibrium pruning by ethnic group using the PLINK. SNPs in high LD (r2≥0.8) retained only a single representative SNP. None of the listed SNPs here were associated with all-ischemic stroke (results not shown) in the GEOS Discovery population. (Table C) PROCR SNP all-ischemic stroke replication results for Caucasians in the young-onset stroke replication cohort: a) without GEOS, and b) with GEOS.
(DOCX)

S1 Dataset Data access. (DOCX)
