# Supplementary material for: Genetics of the thrombomodulin-endothelial cell protein C receptor system and the risk of early-onset ischemic stroke
Source: PLoS One. 2018 Nov 1;13(11):e0206554. doi: 10.1371/journal.pone.0206554 (PMC6211695; doi:10.1371/journal.pone.0206554)
Supplement: S1 File — (Table A) GEOS Characteristics by case–control status. (Table B) Results of linkage-disequilibrium pruning by ethnic group using the PLINK. SNPs in high LD (r2≥0.8) retained only a single representative SNP. None of the listed SNPs here were associated with all-ischemic stroke (results not shown) in the GEOS Discovery population. (Table C) PROCR SNP all-ischemic stroke replication results for Caucasians in the young-onset stroke replication cohort: a) without GEOS, and b) with GEOS. (DOCX) [file pone.0206554.s001.docx]

**Supplementary Information**

**Background**

Thrombomodulin, also known as CD141 and BDCA-3, is an integral membrane protein expressed on the surface of endothelial cells. In humans, thrombomodulin is encoded by the THBD gene (**OMIM 188040 -** <https://omim.org/entry/188040>). THBD maps to chromosome 20p11.2, contains a single exon and no introns, and spans 4 kb. The thrombomodulin protein is expressed primarily on the luminal surface of vascular endothelial cells and consists of 557 amino acids (aa) (60,300 Dalton): an N-terminal lectin-like module (aa 1-154), a hydrophobic region (aa 155-222), six epidermal growth factor (EGF)-like modules (aa 223-462), a serine and threonine rich region (aa 463-497), a single transmembrane segment (aa 498-521), and a short cytoplasmic tail (aa 522-557). Thrombomodulin functions as a cofactor in the thrombin-induced activation of protein C in the anticoagulant pathway by forming a 1:1 stoichiometric complex with thrombin. Binding of thrombomodulin to thrombin suppresses the ability of thrombin to cleave fibrinogen, but enhances >1,000-fold the specificity of the enzyme toward the zymogen protein C. [1] Thrombomodulin-bound thrombin (TT) has no procoagulant effect.

Endothelial protein C receptor, also known as CD 201 and activated protein C receptor (APC receptor), is a protein that in humans is encoded by the PROCR gene (**OMIM 600646 -** <https://omim.org/entry/600646>), which maps to chromosome 20q11.2 and contains 4 exons and spans 6 kb. Sequence analysis predicts a 238-amino acid type 1 transmembrane protein has a 15-amino acid N-terminal signal sequence; an extracellular domain with 4 potential N-glycosylation sites and 4 cys residues; a C-terminal 25-amino acid transmembrane region; and a short cytoplasmic tail containing only 3 amino acids. The protein encoded by this gene is a receptor for activated protein C, a serine protease activated by and involved in the blood coagulation pathway. Binding of protein C to EPCR acts to enhance the activation of protein C. Secondary structure analysis predicted that EPCR folds with a beta-sheet platform supporting 2 alpha-helical regions that collectively form a potential binding pocket for protein C and APC.

**Data**

**GEOS CVD Panel**

The Illumina custom cardiovascular gene-centric 50K SNP array (ITMAT-Broad-CARe array) was implemented on 830 case and 907 control subjects. The fixed panel includes 49,094 SNPs from ~ 2,000 loci, including *THBD* and *PROCR*. Genotyping was performed at the Institute for Translational Medicine and Therapeutics (ITMAT), University of Pennsylvania.

**GEOS GWAS Data**

Samples were genotyped at the Johns Hopkins Center for Inherited Disease Research (CIDR) using the Illumina HumanOmni1-Quad_v1-0_B BeadChip (Illumina, San Diego, CA, USA). Case and control samples were balanced across the plates, and European ancestry (EA) and African ancestry (AA) samples were placed on different plates. Study samples, including 38 blind duplicates chosen from among these samples were plated and genotyped together with 42 HapMap control samples (26 CEPH and 16 Yoruba samples) for quality control, and all samples were processed together in the lab. Allele cluster definitions for each SNP were determined using Illumina BeadStudio Genotyping Module version 3.3.7, Gentrain version 1.0 and the combined intensity data from all released samples. Genotypes were not called if the quality threshold (Gencall score) was below 0.15. Genotypes from 1,827 study individuals, representing 99% of all attempted samples, were released by CIDR, and all SNPs had a genotype call rate > 98%. Overall panel genotyping concordance rate was 99.996% based on study duplicates. We excluded 11 individuals from analysis due to unexpected duplicates, gender discrepancy and unexpected relatedness, leaving a total of 1,816 individuals (889 cases and 927 controls) in the final GWAS sample. A total of 1,014,719 SNPs were released by CIDR, representing 99.83% of all attempted SNPs. Genotypes were not released for SNPs that had call rates less than 85%, a cluster separation value of less than 0.2, more than 1 HapMap replicate error, more than a 5% (autosomal) or 6% (X) difference in call rate between genders, more than 0.3% male AB frequency (X), or more than a 11.3% (autosomal) or 10% (XY) difference in AB frequency. Individual SNPs were excluded post-analysis if they had excessive deviation from Hardy-Weinberg Equilibrium (HWE) proportions (P < 1.0x10-7) or genotype call rates <95%. Departure from HWE was assessed by chi-square test among controls only and within each ethnic group separately. For this report, only SNPs having minor allele frequencies (MAF) > 2% and SNPs passing HWE filtering in both EA and AA populations were included (N = 754,754 SNPs).

**Supplementary Reference**

1. Di Cera E. Thrombin interactions. Chest. 2003 Sep;124(3 Suppl):11S-7S.

<https://www.ncbi.nlm.nih.gov/pubmed/12970119>

**Financial Disclosure and Acknowledgments**

This study was supported in part by NIH grants U01 NS069208, R01 NS100178, and R01 NS105150; an Epidemiology of Aging Training Program Grant, NIH/NIA T32 AG000262; the U.S. Department of Veterans Affairs, and the American Heart Association Cardiovascular Genome-Phenome Study (Grant# 15GPSPG23770000), and an American Heart Association Discovery Grant supported by Bayer Group (Grant# 17IBDG33700328). Further details regarding the data collection, organization, funding and relationships between METASTROKE and the other studies involved can be found below.

**Genetics of Early Onset Stroke (GEOS) Study (Baltimore, USA)**

CVD Chip data for the GEOS Study was supported in part by the National Institutes of Health Genes, Environment and Health Initiative (GEI) Grant U01 HG004436, as part of the GENEVA consortium under GEI; and the Office of Research and Development, Medical Research Service, and the Baltimore Geriatrics Research, Education, and Clinical Center of the Department of Veterans Affairs. Study recruitment and collection of datasets were supported by a Cooperative Agreement with the Division of Adult and Community Health, Centers for Disease Control and by grants from the National Institute of Neurological Disorders and Stroke (NINDS) and the NIH Office of Research on Women's Health (R01 NS45012, U01 NS069208-01). Genotyping of the candidate genes was supported by the American Heart Association (Grant-in-Aid 0855400E) and performed at the University of Maryland Genomics Core with support from the Mid-Atlantic Nutrition and Obesity Research Center (P30 DK072488). GWAS data for the GEOS Study was supported by the National Institutes of Health Genes, Environment and Health Initiative (GEI) Grant U01 HG004436, as part of the GENEVA consortium under GEI, with additional support provided by the Mid-Atlantic Nutrition and Obesity Research Center (P30 DK072488); and the Office of Research and Development, Medical Research Service, and the Baltimore Geriatrics Research, Education, and Clinical Center of the Department of Veterans Affairs. Genotyping services were provided by the Johns Hopkins University Center for Inherited Disease Research (CIDR), which is fully funded through a federal contract from the National Institutes of Health to the Johns Hopkins University (contract number HHSN268200782096C). Assistance with data cleaning was provided by the GENEVA Coordinating Center (U01 HG 004446; PI Bruce S Weir). Study recruitment and collection of datasets were supported by a Cooperative Agreement with the Division of Adult and Community Health, Centers for Disease Control and by grants from the National Institute of Neurological Disorders and Stroke (NINDS) and the NIH Office of Research on Women's Health (R01 NS45012, U01 NS069208-01).

**METASTROKE**

METASTROKE is a collaboration of numerous international studies with the aim of validating associations from previous GWAS and identifying novel genetic associations through meta-analysis of GWAS datasets for ischemic stroke and its subtypes.

Included studies are as follows:

**ASGC**

Australian population control data were derived from the Hunter Community Study. We also thank the University of Newcastle for funding and the men and women of the Hunter region who participated in this study. This research was funded by grants from the Australian National and Medical Health Research Council (NHMRC Project Grant ID: 569257), the Australian National Heart Foundation (NHF Project Grant ID: G 04S 1623), the University of Newcastle, the Gladys M Brawn Fellowship scheme, and the Vincent Fairfax Family Foundation in Australia. Elizabeth G Holliday was supported by a Fellowship from the National Heart Foundation and National Stroke Foundation of Australia (ID: 100071).

**BRAINS**

Bio-Repository of DNA in Stroke (BRAINS) is partly funded by a Senior Fellowship from the Department of Health (UK) to P Sharma, the Henry Smith Charity and the UK-India Education Research Institutive (UKIERI) from the British Council.

*HPS*: Heart Protection Study (HPS) (ISRCTN48489393) was supported by the UK Medical Research Council (MRC), British Heart Foundation, Merck and Co (manufacturers of simvastatin), and Roche Vitamins Ltd (manufacturers of vitamins). Genotyping was supported by a grant to Oxford University and CNG from Merck and Co. Jemma C Hopewell acknowledges support from the British Heart Foundation (FS/14/55/30806).

**ISGS**

Ischemic Stroke Genetics Study (ISGS)/Siblings with Ischemic Stroke Study (SWISS) was supported in part by the Intramural Research Program of the NIA, NIH project Z01 AG-000954-06. ISGS/SWISS used samples and clinical data from the NIH-NINDS Human Genetics Resource Center DNA and Cell Line Repository (http://ccr.coriell.org/ninds), human subjects protocol numbers 2003-081 and 2004-147. ISGS/SWISS used stroke-free participants from the Baltimore Longitudinal Study of Aging (BLSA) as controls. The inclusion of BLSA samples was supported in part by the Intramural Research Program of the NIA, NIH project Z01 AG-000015-50, human subjects protocol number 2003-078. The ISGS study was funded by NIH-NINDS Grant R01 NS-42733 (J F Meschia). The SWISS study was funded by NIH-NINDS Grant R01 NS-39987 (J F Meschia). This study used the high-performance computational capabilities of the Biowulf Linux cluster at the NIH (http://biowulf.nih.gov).

**MGH-GASROS**

MGH Genes Affecting Stroke Risk and Outcome Study (MGH-GASROS) was supported by NINDS (U01 NS069208), the American Heart Association/Bugher Foundation Centers for Stroke Prevention Research 0775010N, the NIH and NHLBI's STAMPEED genomics research program (R01 HL087676), and a grant from the National Center for Research Resources. The Broad Institute Center for Genotyping and Analysis is supported by grant U54 RR020278 from the National Center for Research resources.

**MILANO**

Milano - Besta Stroke Register Collection and genotyping of the Milan cases within CEDIR were supported by the Italian Ministry of Health (Grant Numbers: RC 2007/LR6, RC 2008/LR6; RC 2009/LR8; RC 2010/LR8; GR-2011-02347041). FP6 LSHM-CT-2007-037273 for the PROCARDIS control samples.

**WTCCC2**

Wellcome Trust Case-Control Consortium 2 (WTCCC2) was principally funded by the Wellcome Trust, as part of the Wellcome Trust Case Control Consortium 2 project (085475/B/08/Z and 085475/Z/08/Z and WT084724MA). The Stroke Association provided additional support for collection of some of the St George's, London cases. The Oxford cases were collected as part of the Oxford Vascular Study which is funded by the MRC, Stroke Association, Dunhill Medical Trust, National Institute of Health Research (NIHR) and the NIHR Biomedical Research Centre, Oxford. The Edinburgh Stroke Study was supported by the Wellcome Trust (clinician scientist award to C Sudlow), and the Binks Trust. Sample processing occurred in the Genetics Core Laboratory of the Wellcome Trust Clinical Research Facility, Western General Hospital, Edinburgh. Much of the neuroimaging occurred in the Scottish Funding Council Brain Imaging Research Centre (www.sbirc.ed.ac.uk), Division of Clinical Neurosciences, University of Edinburgh, a core area of the Wellcome Trust Clinical Research Facility and part of the SINAPSE (Scottish Imaging Network—A Platform for Scientific Excellence) collaboration (www.sinapse.ac.uk), funded by the Scottish Funding Council and the Chief Scientist Office. Collection of the Munich cases and data analysis was supported by the Vascular Dementia Research Foundation. M Farrall and A Helgadottir acknowledge support from the BHF Centre of Research Excellence in Oxford and the Wellcome Trust core award (090532/Z/09/Z).

**VISP**

The GWAS component of the Vitamin Intervention for Stroke Prevention (VISP) study was supported by the United States National Human Genome Research Institute (NHGRI), Grant U01 HG005160 (PI Michèle Sale & Bradford Worrall), as part of the Genomics and Randomized Trials Network (GARNET). Genotyping services were provided by the Johns Hopkins University Center for Inherited Disease Research (CIDR), which is fully funded through a federal contract from the NIH to the Johns Hopkins University. Assistance with data cleaning was provided by the GARNET Coordinating Center (U01 HG005157; PI Bruce S Weir). Study recruitment and collection of datasets for the VISP clinical trial were supported by an investigator-initiated research grant (R01 NS34447; PI James Toole) from the United States Public Health Service, NINDS, Bethesda, Maryland. Control data obtained through the database of genotypes and phenotypes (dbGAP) maintained and supported by the United States National Center for Biotechnology Information, US National Library of Medicine.

*WHI*: Funding support for WHI-GARNET was provided through the NHGRI GARNET (Grant Number U01 HG005152). Assistance with phenotype harmonization and genotype cleaning, as well as with general study coordination, was provided by the GARNET Coordinating Center (U01 HG005157). Funding support for genotyping, which was performed at the Broad Institute of MIT and Harvard, was provided by the NIH Genes, Environment, and Health Initiative (GEI; U01 HG004424).

**SiGN**

The Stroke Genetics Network (SiGN) study was funded by a cooperative agreement grant from the National Institute of Neurological Disorders and Stroke (NINDS) U01 NS069208. Genotyping services were provided by the Johns Hopkins University Center for Inherited Disease Research (CIDR), which is fully funded through a federal contract from the National Institutes of Health (NIH) to the Johns Hopkins University (contract No. HHSN268200782096C). The Biostatistics Department Genetics Coordinating Center at the University of Washington (Seattle) provided more extensive quality control of the genotype data through a subcontract with CIDR. Additional support to the Administrative Core of SiGN was provided by the Dean’s Office, University of Maryland School of Medicine. This work was supported by grants received from the German Federal Ministry of Education and Research (BMBF) in the context of the e:Med program (e:AtheroSysMed), the FP7 European Union project CVgenes@target (261123), the DFG as part of the CRC 1123 (B3), the Corona Foundation and the Foundation Leducq (Transatlantic Network of Excellence on the Pathogenesis of Small Vessel Disease of the Brain).

**Supplementary Tables**

**Supplementary Table A**: GEOS Characteristics by case–control status

|  | **Cases (*n* = 829)** | **Controls (*n* = 850)** | ***P*-value** |
| --- | --- | --- | --- |
| Mean age (years) | 41.1 ± 6.9 | 39.6 ± 6.8 | <0.0001 |
| Female (%) | 41.8 | 43.3 | 0.55 |
| Self-Reported Race (%)  European Ancestry (EA)  African Ancestry (AA)  Other | 52.8  42.2  5.1 | 56.6  38.1  5.3 | 0.22 |
| Hypertension (%) | 43.1 | 19.1 | <0.0001 |
| Diabetes mellitus (%) | 16.9 | 5.2 | <0.0001 |
| Current smokers (%) | 42.1 | 28.3 | <0.0001 |
| Angina/MI (%) | 5.5 | 0.7 | <0.0001 |
| ≥ 1 Vascular Risk Factor* (%) | 69.2 | 43.1 | <0.0001 |

**Vascular risk factors: hypertension, diabetes mellitus, current smoking, angina/MI*

**Supplementary Table B**: Results of linkage-disequilibrium pruning by ethnic group using the PLINK. SNPs in high LD (r^2^≥0.8) retained only a single representative SNP. None of the listed SNPs here were associated with all-ischemic stroke (results not shown) in the GEOS Discovery population.

| **Ethnicity-Gene,**  **rs-number, alleles** | **Position** | **GWAS and/or**  **CVD Chip** | **SNPs in LD (r^2^>0.8)** |
| --- | --- | --- | --- |
|  |  |  |  |
| **AA - PROCR** |  |  |  |
| rs2069941, T/C | 33223047 | GWAS | NA |
| rs7265317, C/T | 33232184 | GWAS | SNP20-33231431 |
| rs9574, C/G | 33764632 | GWAS | SNP20-33226515, SNP20-33227612, rs2378337, rs1415774 (GWAS and CVD), rs2065979, SNP20-33231913, SNP20-33238105 |
| rs8119351, A/G | 33218066 | GWAS/CVD Chip | NA |
| rs6088747, G/T | 33218265 | GWAS/CVD Chip | NA |
| rs867186, G/A | 33228215 | GWAS/CVD Chip | NA |
| rs6087682, T/A | 33216558 | GWAS | SNP20-33216923 |
| rs1415775, T/C | 33229432 | GWAS | NA |
| SNP20-33226893, G/A | 33226893 | GWAS | NA |
| rs2069951, A/G | 33227425 | GWAS | NA |
| rs73903026, A/G | 33233416 | GWAS | SNP20-33225711 |
|  |  |  |  |
| **AA -THBD** |  |  |  |
| rs2567609 | 22965017 | GWAS | NA |
| rs6048519 | 22982274 | GWAS | NA |
| rs1042579 | 22976724 | GWAS | rs3176123 |
| SNP20-22965082 | 22965082 | GWAS | NA |
| SNP20-22964970 | 22964970 | GWAS | rs6076013 |
| rs1042580 | 22975621 | GWAS | NA |
| rs1962 | 22974496 | GWAS | NA |
| rs2424505 | 22984049 | GWAS | NA |
| rs2007363 | 22973295 | GWAS | NA |
| rs3176122 | 22975917 | GWAS | NA |
| rs2567607 | 22965507 | GWAS | NA |
| rs8123616 | 22982556 | GWAS | NA |
| rs1883957 | 22968003 | GWAS | NA |
|  |  |  |  |
| **EA -THBD** |  |  |  |
| rs2567609 | 22965017 | GWAS | SNP20-22965082 |
| rs4988478 | 22965294 | GWAS | NA |
| rs2567607 | 22965507 | GWAS | NA |
| rs1883957 | 22968003 | GWAS | NA |
| rs6076013 | 22969597 | GWAS | SNP20-22964970 |
| rs2007363 | 22973295 | GWAS | NA |
| rs3176126 | 22974271 | GWAS | NA |
| rs1962 | 22974496 | GWAS | NA |
| rs3176123 | 22975413 | GWAS | rs1042579 |
| rs1042580 | 22975621 | GWAS | NA |
| rs6048519 | 22982274 | GWAS | NA |
| rs8123616 | 22982556 | GWAS | NA |
| rs2424505 | 22984049 | GWAS | NA |
|  |  |  |  |
| rs1040585 | 22988066 | CVD Chip | NA |
| rs2007743 | 22987041 | CVD Chip | rs6076016, rs2424503 |
| rs13038379 | 22984741 | CVD Chip | rs2896939 |
| rs6082986 | 22971919 | CVD Chip | NA |

**Supplementary Table C:** *PROCR* SNP all-ischemic stroke replication results for Caucasians in the young-onset stroke replication cohort: a) without GEOS, and b) with GEOS.

|  |  | **Young-Onset Stroke Replication Cohort**  **without GEOS EA (6 studies)** | | | | | **Young-Onset Stroke Replication Cohort**  **with GEOS EA (7 studies)** | | | | |
| --- | --- | --- | --- | --- | --- | --- | --- | --- | --- | --- | --- |
| SNP | Reference Allele Bolded | Cases/Controls | EAF | Beta | SE | P-Value | Cases/Controls | EAF | Beta | SE | P-Value |
| rs9574 | **C**/G | 3671/21119 | 0.397 | 0.077 | 0.033 | **0.015** | 4119/21617 | 0.399 | 0.117 | 0.049 | **0.017** |
| rs6087682 | **A**/T | 3671/21119 | 0.766 | 0.034 | 0.038 | 0.368 | 4119/21617 | 0.765 | 0.060 | 0.046 | 0.195 |
| rs2069951 | **G**/A | 3671/21119 | 0.956 | 0.079 | 0.081 | 0.331 | 4119/21617 | 0.953 | 0.187 | 0.134 | 0.163 |
| rs1415775 | **C**/T | 3671/21119 | 0.759 | 0.012 | 0.037 | 0.745 | 4119/21617 | 0.756 | 0.002 | 0.035 | 0.963 |
| rs867186 | **A**/G | 3671/21119 | 0.865 | 0.103 | 0.069 | 0.133 | 4119/21617 | 0.870 | 0.109 | 0.049 | 0.025 |
