# Supplementary material for: Genetics of the thrombomodulin-endothelial cell protein C receptor system and the risk of early-onset ischemic stroke
Source: PLoS One. 2018 Nov 1;13(11):e0206554. doi: 10.1371/journal.pone.0206554 (PMC6211695; doi:10.1371/journal.pone.0206554)
Supplement: S1 Dataset — (DOCX) [file pone.0206554.s002.docx]

**Data Access**

The data used in this study was collected independently by the studies and Institutions as listed in the manuscript and supplementary information file. Each study has different data sharing policies. For the most part, each study can be contacted to attain their data, and for NIH funded studies, the study data is available via database of Genotypes and Phenotypes (dbGaP) @ <https://www.ncbi.nlm.nih.gov/gap/>. As described in the manuscript’s Methods section, the aggregated data used in this study are available from the corresponding author and participating studies upon reasonable request. As stated, each study can be contacted to request their data individually via the following emails:

The Genetics of Early Onset Stroke (GEOS) Study: [jcole@som.umaryland.edu](mailto:jcole@som.umaryland.edu) and/or [skittner@umaryland.edu](mailto:skittner@umaryland.edu)

Cervical Artery Dissections and Ischemic Stroke Patients (CADISP): [sdebette@bu.edu](mailto:sdebette@bu.edu)

<https://www.cadisp.com/topic/index.html>

MILANO (from Besta Cerebrovascular Diseases Registry): [Giorgio.Boncoraglio@istituto-besta.it](mailto:Giorgio.Boncoraglio@istituto-besta.it)

Risk Assessment of Cerebrovascular Events (RACE): [saleheen@mail.med.upenn.edu](mailto:saleheen@mail.med.upenn.edu)

Stroke in Young Fabry Patients (SIFAP): [arndt.rolfs@med.uni-rostock.de](mailto:arndt.rolfs@med.uni-rostock.de)

<http://www.sifap.de/>

Wellcome Trust Case-Control Consortium 2 (WTCCC2): [cathie.sudlow@ed.ac.uk](mailto:cathie.sudlow@ed.ac.uk)

METASTROKE: [Rainer.Malik@med.uni-muenchen.de](mailto:Rainer.Malik@med.uni-muenchen.de)

The Stroke Genetics Network (SiGN): [jcole@som.umaryland.edu](mailto:jcole@som.umaryland.edu) and/or [skittner@umaryland.edu](mailto:skittner@umaryland.edu)
